# Spinal Deformities after Childhood Tumors

**DOI:** 10.3390/cancers12123555

**Published:** 2020-11-28

**Authors:** Anna K. Hell, Ingrid Kühnle, Heiko M. Lorenz, Lena Braunschweig, Katja A. Lüders, Hans Christoph Bock, Christof M. Kramm, Hans Christoph Ludwig, Konstantinos Tsaknakis

**Affiliations:** 1 Pediatric Orthopedics, Department of Trauma, Orthopedic and Plastic Surgery, University Medical Center Göttingen; 37075 Göttingen, Germany; heiko.lorenz@med.uni-goettingen.de (H.M.L.); lena.braunschweig@med.uni-goettingen.de (L.B.); katja.lueders@med.uni-goettingen.de (K.A.L.); konstantinos.tsaknakis@med.uni-goettingen.de (K.T.); 2 Division of Pediatric Hematology and Oncology, University Medical Center Göttingen; 37075 Göttingen, Germany; ingrid.kuehnle@med.uni-goettingen.de (I.K.); christof.kramm@med.uni-goettingen.de (C.M.K.); 3 Department of Neurosurgery, Division of Pediatric Neurosurgery, University Medical Center Göttingen, 37075 Göttingen, Germany; christoph.bock@med.uni-goettingen.de (H.C.B.); hludwig@med.uni-goettingen.de (H.C.L.)

**Keywords:** spinal deformity, central nervous system, tumor, children, treatment, intramedullary spinal cord tumor

## Abstract

**Simple Summary:**

A significant number of children surviving intra- or juxta-spinal tumors develop secondary spinal deformities and disabilities. This retrospective non-comparative study focuses on deformity analysis, age, and skeletal maturity dependent treatment options and results. Patients who developed severe scoliosis, pathological kyphosis, and/or lordosis were either treated conservatively or surgically by using growth-friendly spinal implants in younger children or definite spinal fusion during puberty. Despite severe spinal deformity, some patients were not surgically corrected in order to preserve mobility through trunk motion or malignant tumor progression. Growth-friendly spinal implants and spinal fusions were able to significantly reduce pathological curves. The first method, with bilateral rib-to-pelvis fixation, still allows spinal magnetic resonance imaging or neurosurgical intervention if needed. Severe ad hoc curve correction enhances the risk of neurological deterioration because of prior spinal cord impairment. The data of this study may help to improve the individual patient care.

**Abstract:**

Childhood tumors of the central nervous system (CNS) and other entities affecting the spine are rare. Treatment options vary from surgical biopsy to partial, subtotal, and total resection, to radiation, to chemotherapy. The aim of this study is to investigate spinal deformity and subsequent surgical interventions in this patient cohort. A retrospective review at our institution identified children with CNS tumors, spinal tumors, and juxta-spinal tumors, as well as spinal deformities. Tumor entity, treatment, mobilization, and radiographic images were analyzed relative to the spinal deformity, using curve angles in two planes. Conservative or surgical interventions such as orthotic braces, growth-friendly spinal implants, and spinal fusions were evaluated and analyzed with respect to treatment results. Tumor entities in the 76 patients of this study included CNS tumors (*n* = 41), neurofibromatosis with spinal or paraspinal tumors (*n* = 14), bone tumors (*n* = 12), embryonal tumors (*n* = 7), and others (*n* = 2). The initial treatment consisted of surgical biopsy (*n* = 5), partial, subtotal, or total surgical resection (*n* = 59), or none (*n* = 12), followed by chemotherapy, radiotherapy, or both (*n* = 40). Out of 65 evaluated patients, 25 revealed a moderate or severe scoliotic deformity of 71° (range 21–116°), pathological thoracic kyphosis of 66° (range 50–130°), and lordosis of 61° (range 41–97°). Surgical treatment was performed on 21 patients with implantation of growth-friendly spinal implants (*n* = 9) as well as twelve dorsal spinal fusions (two with prior halo distraction). Surgical interventions significantly improved spinal deformities without additional neurological impairment. With the increasing number of children surviving rare tumors, attention should be focused on long-term problems such as spinal deformities and consequent disabilities. A significant number of children with CNS tumors, spinal tumors or juxta-spinal tumors required surgical intervention. Early information about spinal deformities and a close follow-up are mandatory for this patient group.

## 1. Introduction

Spinal central nervous system (CNS) tumors, bone or embryonal tumors, neurofibromatosis, metastasis of vertebral bodies, and thoracic malignancies are rare diagnoses in the pediatric population. Complete tumor elimination without damaging the surrounding spinal cord permanently is the main purpose of the treatment. Although survival rate for childhood tumors has continuously increased in previous decades, tumors involving the spine or spinal cord often impair neurological function, spinal development, or walking ability.

Progressive spinal deformity as a result of decompression surgery, laminectomy, radiation therapy, or the tumor itself is a well-described risk in cases with spinal tumors [1,2,3,4,5]. The severity of the deformity depends on the age of the patient, the number of surgically involved vertebrae, laminectomy or laminoplasty, and the affected area of the spine [2,3,6]. In addition, progressive spinal deformity in childhood can lead to limited cardiopulmonary functions known as thoracic insufficiency syndrome [7] and often requires early surgical interventions to prevent a further progression of deformity [8]. This can prove quite challenging, as spinal implants can interfere with magnetic resonance imaging (MRI) to assess CNS tumor progression. Furthermore, the neurosurgical approach needs to be preserved for eventual secondary CNS tumor surgery in the future. Using growth-friendly spinal implants such as the vertical expandable prosthetic titanium rib (VEPTR), devices parallel to the spine with a rib-to-pelvis fixation [8,9,10,11,12] respect such needs, allowing access to the spinal canal and MRI. Long-term results of this technique on young paraplegic children with intraspinal tumors, although less favorable in comparison to its application on neuromuscular scoliosis due to spinal muscular atrophy, have been described by Lorenz et al. [11].

Definite spinal fusion is usually performed at puberty to avoid curve progression and for deformity correction [2]. For advanced spinal curves that are combined with an impaired spinal cord after malignancy, the neurological risks at surgical correction are enhanced [2]. There is a high risk of neurological complications during surgical correction of severe scoliotic curves due to a tumor-induced impairment of spinal cord function. The possible risks of spinal cord injuries include any developing myelopathies caused by severe spinal deformity, hypomochlion-like structures, and tethering. All these problems should be strictly avoided because chronic spinal cord lesions might occur, which perpetuate even later after correction surgery. Therefore, the development of severe spinal deformities in this challenging patient group should be avoided through early intervention [13].

This retrospective, non-comparative study evaluates spinal deformities after childhood spinal tumors. Different treatment options, subsequent problems, and surgical results are presented and discussed.

## 2. Results

The study included 76 pediatric patients (45 male and 31 female) who had a primary diagnosis of spinal or juxta-spinal tumor at an age ≤ 18 years (Table 1).

**Figure 1:**
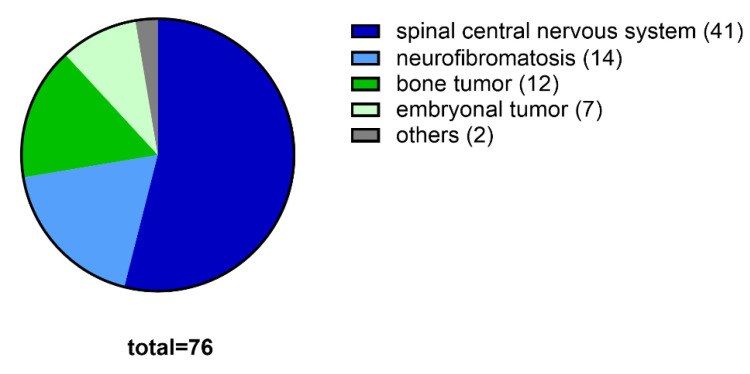
Tumor entities of the studied pediatric population (*n* = 76).

The included diagnoses were spinal CNS tumors (*n* = 41, 54%), neurofibromatosis (*n* = 14, 18%), bone tumors (*n* = 12, 16%), embryonal tumors (*n* = 7, 9%), and others (*n* = 2, 3%) (Figure 1). Spinal CNS tumors were high-grade in 34% (*n* = 14) and low-grade in 66% (*n* = 27) of cases. Bone tumors included osteoid osteoma, aneurysmal bone cyst, Ewing sarcoma, and spinal osteosarcoma. Embryonal tumors were mainly neuroblastoma, whereas other tumor entities were sarcoma.

Out of this group, 64 children (84%) had a surgical intervention after the initial diagnosis. The mean age at the time of surgery was 9.6 years (SD = 5.9; range 0–18.9) years. The goal of the initial intervention was either confirmation of diagnosis with a biopsy (*n* = 5) or partial, subtotal, or total tumor resection (*n* = 59). A combination of chemotherapy and radiotherapy was applied in 27 cases (36%), whereas nine children were treated with chemotherapy alone (12%) and four patients with only radiotherapy (5%). Twenty-seven children (36%) had no chemotherapy or radiotherapy. At the time of evaluation, 60 were ambulatory (79%) and 16 were wheelchair dependent (21%). Fifteen patients (20%) died because of their malignancy.

Radiological images were performed in 65 children and adolescents (86%) for spinal deformity assessment. Forty of these patients (62%) had no spinal deformity (*n* = 31) or mild deformity (*n* = 9) which required no treatment. Four patients (6%) had moderate spinal deformity with an average of 28° (SD = 6.3; range 21–36°), whereas 21 patients were diagnosed with severe deformity, the main curve angle of which averaged at 80° (SD = 21.9; range 42–116°).

Physiological kyphosis within the normal range of variation [14] was seen in 16 cases with an average angle of 24° (SD = 7.5; range 15–37°). Pathologic kyphosis occurred in 24 patients with an average kyphosis angle of 66° (SD = 20.1; range 50–130°). Normal lordosis patients (*n* = 21) had an average curve degree of 24° (SD = 11.2; range 4–38°). Pathologic lordosis beyond the normal range of variation [15] was obvious in 13 patients with an average lordosis angle of 61° (SD = 14.6; range 41–97°).

**Figure 2:**
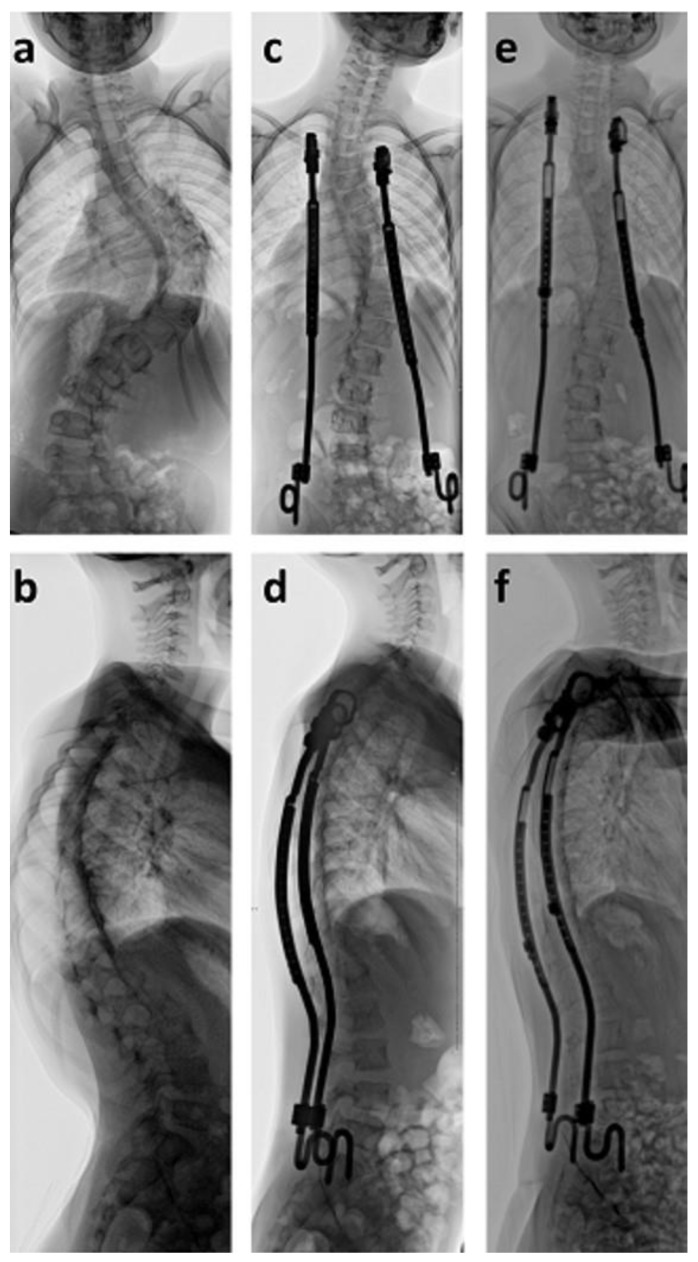
Seven-year old girl with neurofibromatosis type I and scoliosis of 101° (**a**,**b**). Bilateral rib-to-pelvis vertical expandable prosthetic titanium rib (VEPTR) devices were able to correct the scoliotic deformity to 43° (**c**,**d**). VEPTR implants are surgically lengthened every six months (**e**,**f**) to maintain the correction and to permit growth.

Surgical deformity correction was performed with bilateral VEPTR devices implanted in children ten years or younger. Using this technique, nine cases were treated and received an average of nine lengthening procedures every six months so far. As a result, the scoliotic curve was significantly (*p* < 0.001) corrected from an average of 83° to 49° (41%) directly after surgical implantation (Figure 2). Kyphosis significantly (*p* = 0.0133) improved from 60° to 38°, whereas lordosis remained stable at 32°. A careful evaluation of CNS structures was performed prior to surgical spinal deformity correction. This included MRI, neurosurgical evaluation, and analysis of evoked potentials in order to evaluate the risk of neurological complications. There was no neurological deterioration in the treated group.

**Figure 3:**
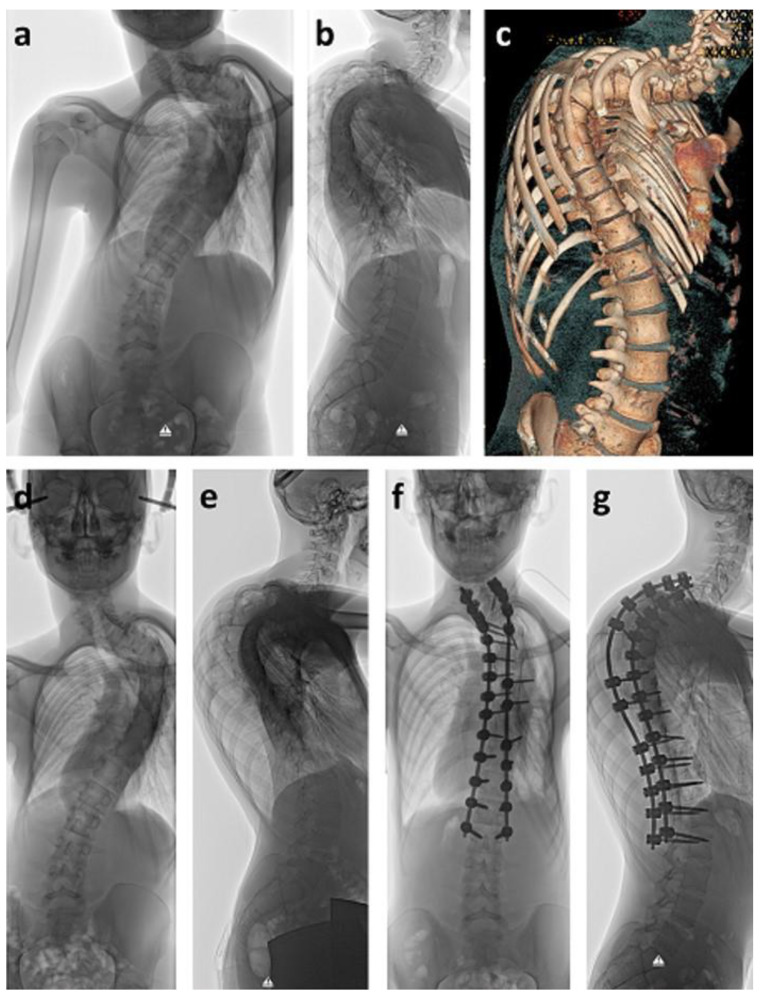
Fifteen-year old boy who was diagnosed with intraspinal astrocytoma at age ten. He received chemotherapy and radiotherapy. Five years later, severe thoracic spinal deformity had developed (**a**–**c**). Halo traction was applied for gradual scoliosis and kyphosis correction as well as reduction of the surgical risk due to neurological deterioration (**d**,**e**). Surgery was performed using spinal cord monitoring. When erecting the spine, motor evoked potentials (MEP) disappeared. After controlling correct screw placement by computed tomography, spinal deformity correction could only be performed until MEP weakened, thus leaving considerate kyphosis as a residual deformity (**f**,**g**).

During puberty, twelve patients with spinal deformities received a definite spinal fusion. Reasons were scoliosis alone (*n* = 2), kyphosis alone (*n* = 2), or a three-dimensional deformity (*n* = 8). In two cases with severe, high, and rigid thoracic deformities of 108° and 105° (Figure 3), halo traction was applied for eight weeks prior to surgical intervention, in order to reduce the deformity through gradual correction. No neurological impairment occurred during halo traction. Despite thorough pre-surgical and intra-surgical planning and spinal cord monitoring, loss of motor function potential did occur in these severe cases during surgery. After ruling out screw misplacement by CT scans, deformity correction was reduced to a point of compromise between the best possible alignment and full motor function potential (Figure 3).

The significant (*p* = 0.0026) average scoliotic curve correction of the spinal fusion group (*n* = 12) was from 66° to 32° (51%). Kyphosis significantly (*p* < 0.001) improved from 75° to 47°, whereas lordosis remained identical.

## 3. Discussion

Childhood tumors of the CNS, the spine, and the thorax may lead to spinal deformity. In the literature, incidence rates for scoliosis after spinal tumors varies between 8 and 88% [5,15,16]. In our study population, radiological evaluation of the spine was obtained in 86% of the patients, and out of these, about one third (38%) developed moderate to severe three-dimensional spinal deformity. Radiological follow-up of the spine was only performed in children with developing spinal deformities or where it was suspected that incorrect growth had started. Therefore, 14% of patients were not radiologically evaluated but kept in the study population for a more realistic perspective. Causes for the development of scoliosis, pathological kyphosis, or lordosis after spinal tumors in children are multifactorial. One of the main problems leading to spinal deformity is spinal column instability after laminectomy or laminoplasty, even though the latter method seems to be more favorable [17]. Unfavorable results seem to be caused by a four or more level laminectomy, as well as its location in the thoracolumbar junction [2]. Additional factors are initial tumor grading, radiotherapy, a relapse of a tumor, secondary malignancy after treatment, chemotherapy with adverse effects on bone mineral density, and secondary osteoporosis [15,17,18,19,20]. It is well understood that younger children are more prone to develop progressive deformities due to the remaining growth potential of an immature skeleton [13,20,21].

Additionally, in our patient group, childhood tumor survivors were often presented late with severe deformities in comparison to idiopathic or neuromuscular scoliosis patients, probably because five-year survival after tumor surgery was the main objective. Neurological impairment also plays an important role in the development of spinal deformities. The trigger for the development of scoliosis, pathological kyphosis, or lordosis lies in the neuromuscular imbalance as seen in non-ambulatory children with neuromuscular disease (e.g., Duchenne muscle dystrophy, spinal muscular atrophy, and others) [22].

Despite their having a spinal deformity, some neurologically impaired but walking patients did not receive surgical intervention at that time in order to preserve mobility. In children with myelomeningocele it is well known that motion amplitudes of the trunk are related to the degree of muscle weakness of the lower limbs [23]. Baniasad et al. [24] explored the significance of trunk and upper extremity muscles in the paraplegic gait. From integrating these findings into an individual treatment concept, surgical intervention may be cancelled or postponed in order to preserve function and independence rather than correct statics.

Orthotic brace treatment is often unsuccessful in neuromuscular scoliosis and tumor-induced spinal deformities [25]. Braces can be used to achieve trunk stability but usually do not sufficiently correct pathological deformity in these patients. Again, a spine brace might negatively influence walking in neurologically compromised patients. However, if severe curve progression was noted in marginal ambulatory, neurologically impaired children, brace treatment was performed during sitting activity (e.g., school) to halter curve progression. The numbers in this paper are too small to draw significant conclusions and to prove whether this was a successful strategy.

Many studies have proven the efficiency of several growth-friendly spinal implants with various fixation and distraction methods to control spinal deformity and allow thoracic growth [26,27,28]. Bilateral rib-to-pelvis VEPTR constructs have the advantage of leaving the spine untouched as well as being expanded without the use of magnetic components, thus allowing MRI examinations after device implantation [10]. In our study, nine skeletally immature patients received VEPTR treatment with an average deformity correction of 41% after initial implantation, which lies within the range of 29–41% correction as reported in other studies [11,12,27]. A small group of paraplegic children with intraspinal tumors who were treated with bilateral growth-friendly spinal implants had been previously analyzed and compared to neuromuscular patients by our study group [11]. Even though these patient numbers are small, a significant initial curve correction constantly showed more severe curve progression over time in the tumor group and less favorable pelvic obliquity control [11].

In adolescents with progressive or severe spinal deformity, definite spinal fusion is the treatment of choice. In this study, the incidence of spinal fusion after spinal childhood tumors was 16% (*n* = 12), which is within the literature’s described range of 15 to 27% [1,2]. Due to spinal cord alteration that occurs either post-radiation or because of scar tissue from surgery, the neurological risk from surgical correction is enhanced. Therefore, some authors [2,13,21] have analyzed and postulated the effects of preventative spinal fusions in order to avoid progressive deformities later. Yao et al. identified in age < 13 years preoperative spinal deformity, involvement of the thoracolumbar junction, and tumor-associated syrinx as well as multiple tumor resections as independent risk factors for the development of spinal deformity after intramedullary spinal cord tumors [2]. They postulated preventative spinal fusion. However, this therapeutic concept was heavily criticized by others [29]. Possible other risks of spinal cord injuries include any developing myelopathies caused by severe spinal deformity, hypomochlion-like structures, and tethering. All these problems can cause chronic spinal cord lesions, which may even perpetuate later after correction surgery.

The limitations of this retrospective, non-comparative, single-center study are that the patients were recruited by three different medical specialties and presented for different reasons (e.g., primary tumor diagnosis and treatment, surgical treatment of spinal cord malignancies, and treatment of spinal deformity). Additionally, the lack of a prospective standardized evaluation at the time of initial diagnosis, directly after intramedullary surgery and during follow-up, make a valid comparison difficult. The multivariate analysis by Yao et al. identifying risk factors for the development of spinal deformities which required spinal fusion in children with intramedullary spinal cord tumors was controversially criticized by Angevine and McCormick [2,29]. They discussed that a retrospective, non-comparative, single-center study may provide confirmatory evidence of previously noted associations, but that statistically valid conclusions would be problematic. The same criteria can be applied to our investigation. A prospective, multi-center, nationally or internationally based registry study with a prospective follow-up design focusing on spinal deformity development after spinal, intra-spinal, and juxta-spinal spinal tumors in children would answer many questions, such as the overall incidence of spinal deformity, and dependent and independent confounding parameters. To our knowledge, however, such investigations have not been performed so far. Therefore, we nevertheless feel that the presentation of treatment options and results for these rare childhood tumors may help to improve individual patient care. A sharpening of awareness for this problem will most likely help to detect scoliosis earlier, thus preventing severe and more difficult to treat spinal deformities.

## 4. Materials and Methods

After approval was obtained by the institutional ethical review committee of the University Medical Center Goettingen (DOK_125_2013, obtained 5 December 2013), we performed a retrospective single-center analysis of children and adolescents with childhood tumors affecting the spine, spinal canal, or paraspinal structures. The ethics committee waived the need for informed consent for the study.

Patients were recruited either through (a) the Pediatric Hematology and Oncology department of our university, (b) the Pediatric Neurosurgery unit for surgical treatment of CNS tumors, or (c) the Pediatric Orthopedic Surgery for secondary treatment of spinal deformity. The majority of patients were treated by several units. All patients in the study received a proper tumor workup.

Charts, tumor board recommendations, and radiographic investigations were reviewed for all patients, and demographic, clinical, radiological, surgical, histopathological, and follow-up data were collected.

**Figure 4:**
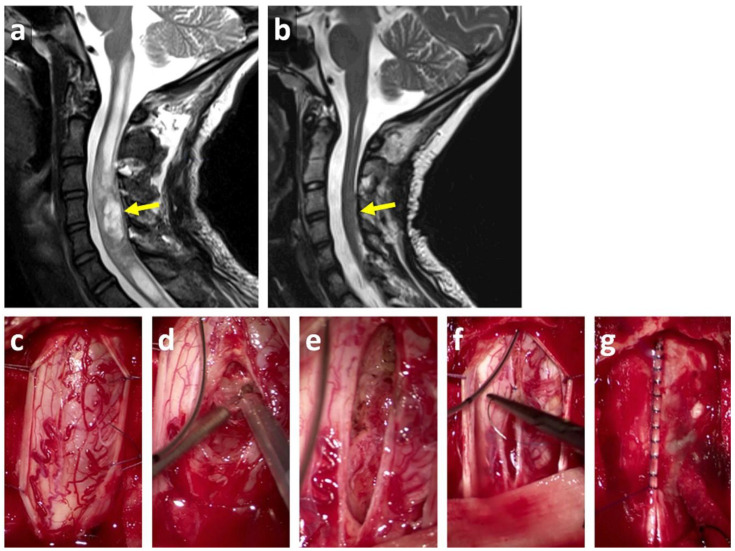
MRI presentation of a cervical pilocytic astrocytoma grade I of the World Health Organization (WHO) grading system (yellow arrow) before (**a**) and after surgical resection (**b**) in a 14-year old girl. Surgical situs shows opening of the dura (**c**), CUSA (cavitronic ultrasonic surgical aspirator) de-bulking (**d**), opened cavitying of the spinal cord with partially removed tumor (**e**), closure of the spinal cord (**f**) and dural closure (**g**).

Patients were grouped using the following entities—low-grade or high-grade spinal CNS tumors, bone tumors, embryonal tumors, neurofibromatosis associated with spinal or paraspinal tumors, and others. Initial treatment was analyzed and consisted mainly of biopsy, partial, subtotal, or total neurosurgical resection (Figure 4), followed by either chemotherapy, radiotherapy, or both; or no treatment at all. At follow-up, spinal deformities, mobilization, and mortality were analyzed. Radiological follow-up of the spine was only performed in children with developing spinal deformity or the suspicion that incorrect growth had started.

**Figure 5:**
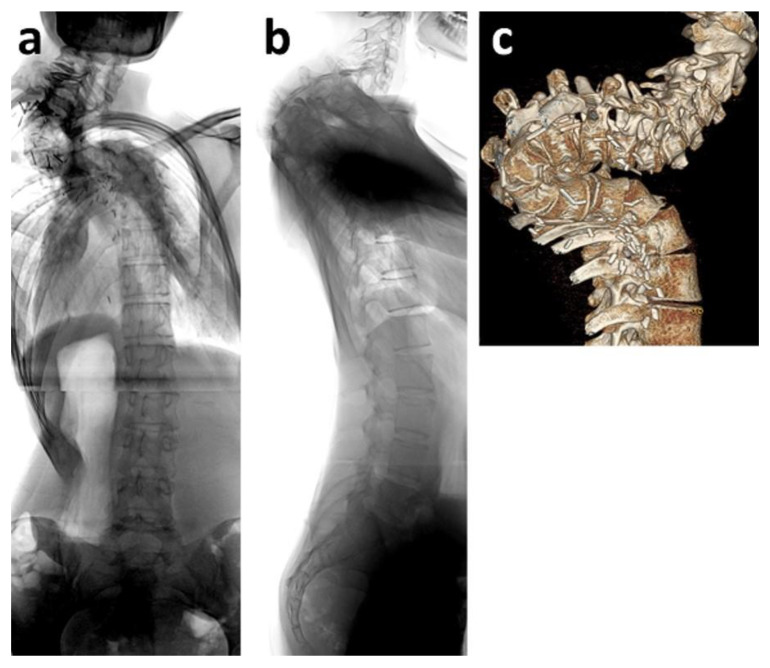
Fifteen-year old girl with juxta-spinal neurofibromatosis and severe three-dimensional upper thoracic spinal deformity (**a**–**c**). MRI prior to surgical treatment revealed severe neurofibromatosis with thoracic obstruction, which was incompletely removed.

After radiological investigation, spinal deformity was divided into scoliosis, kyphosis, and lordosis. Each quantification of curves was done using the Cobb angle [30]. Scoliosis was defined as not existent (curve angle < 10°), mild (10–20°), moderate (21–40°), and severe (>40°). Thoracic kyphosis and lumbar lordosis are highly variable in children and are age dependent [14]. Values that were graded as pathological and were additionally quantified were those that were beyond the normal range of variation [14], i.e. above 40°, and those where abnormal curve patterns occurred such as kyphosis within the lumbar region. Radiographic evaluations were performed using standing or sitting a.p. (anterior posterior), lateral radiographs (Figure 5), MRI, or computed tomography (CT) scans if needed.

Spinal deformities were assessed before and after surgical treatment in cases that were indicated for surgical spinal deformity correction. The surgical treatment consisted either of an implantation of growth-friendly spinal devices with repetitive lengthening procedures in children younger than ten years of age so as to allow spinal and thoracic growth or a definite spinal fusion during puberty, with or without prior halo traction, to stretch and straighten the spine and to reduce neurological risks.

Growth-friendly spinal implants such as bilateral vertical expandable prosthetic titanium ribs (VEPTR) were implanted with a rib-to-pelvis fixation [8,9,10,11,12]. This spinal non-touch technique still allowed MRI assessment of spinal tumors and neurosurgical intervention if needed. Indication for definite spinal fusion was a severe curve progression, spinal instability, and a minimum age of 10 years. In cases with less progression and milder curves, spinal fusion was performed ideally after reaching adequate spinal length and towards the end of skeletal growth measured by Risser sign > 2 [31].

**Figure 6:**
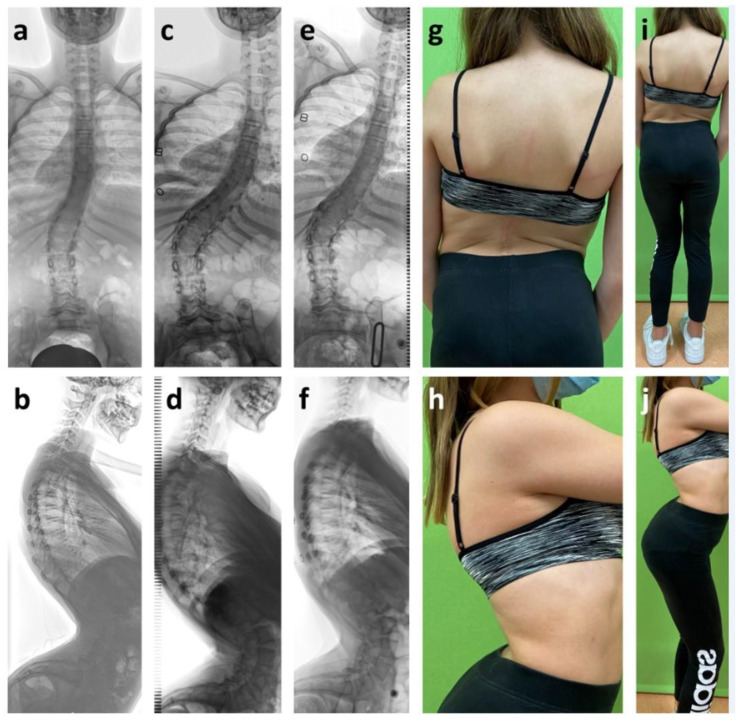
Girl with intramedullary glioma WHO I° from T8 to L3, which was initially diagnosed at age two and partially removed using laminoplasty. At age 12, the patient had mild scoliosis and hyperkyphosis as well as severe lordosis (**a**,**b**). At age 14, deformity had progressed to 53° scoliosis, 60° kyphosis and 97° lordosis (**c**,**d**). Brace therapy did not correct the deformity sufficiently (**e**,**f**). Clinical presentation (**g**–**j**) reveals hip flexion contractures and enhanced lumbar lordosis. The patient needed trunk motion for walking due to a neurological impairment after treatment.

A subgroup of patients did not undergo surgical spinal deformity correction, either because of unknown tumor survival, relapse, or in the case of barely ambulatory patients. The latter group had impaired neurological function but were still able to walk through compensating trunk motion. In these cases, spinal fusion after surgical treatment would most probably result in the loss of walking ability. However, if progressive spinal deformity was observed for these patients, they were treated with conservative braces instead of a surgery, mainly during sitting activities (e.g., school). Curve correction during brace treatment was analyzed as well (Figure 6).

The obtained data were reviewed statistically with a paired Student’s *t*-test using Excel (Microsoft Corporations, Redmond, CA, USA). All data are presented as mean ± standard deviation. Statistical significance was determined as *p* < 0.05.

## 5. Conclusions

After spinal, intra-spinal, or juxta-spinal childhood tumors, a significant proportion of children develop spinal deformity. Conservative treatment options vary from none, observation alone, to orthotic brace therapy, which is usually not successful. In young children, growth-friendly spinal implants significantly correct scoliosis, as well as definite spinal fusion during puberty. A careful evaluation of neurological deficits and abilities is mandatory to apply the best individual care. A prospective clinical and radiological evaluation for spinal deformities, along with the assessment of skeletal maturity, postoperative serial clinical evaluations, and, if necessary, radiological evaluations, will most likely improve individual treatment options and avoid severe, neurologically challenging deformities.

## Figures and Tables

**Table 1:** Overview of patient characteristics.

Variable	Value
Number of Patients	76
female	31
male	45
Tumor Type	
spinal CNS (high-grade/low-grade)	41 (14/27)
neurofibromatosis with spinal or paraspinal tumors	14
bone tumor	12
embryonal tumor	7
others	2
Initial Surgical Tumor Treatment	
biopsy	5
partial, subtotal or total resection	59
none	12
Malignancy Treatment	
chemotherapy	9
radiotherapy	4
combination chemo- and radiotherapy	27
none	37
Spinal Deformity (*n* = 65)	
Scoliosis	
none (≤10°)	31
mild (>10°; ≤20°)	9
moderate (>20°; ≤40°)	4
severe (>40°)	21
Kyphosis (*n* = 40)	
within normal range^15^	16
pathological	24
Lordosis (*n* = 34)	
within normal range^15^	21
pathological	13
Treatment of Spinal Deformity	
none (or brace for stability)	44
growth-friendly spinal implants	9
spinal fusion	12

## References

[1] Hersh D.S., Iyer R.R., Garzon-Muvdi T., Liu A., Jallo G.I., Groves M.L. (2017). Instrumented fusion for spinal deformity after laminectomy or laminoplasty for resection of intramedullary spinal cord tumors in pediatric patients. Neurosurg. Focus.

[2] Yao K.C., McGirt M.J., Chaichana K.L., Constantini S., Jallo G.I. (2007). Risk factors for progressive spinal deformity following resection of intramedullary spinal cord tumors in children: An analysis of 161 consecutive cases. J. Neurosurg. Pediatr..

[3] Papagelopoulos P.J., Peterson H.A., Ebersold M.J., Emmanuel R.P., Choudhury S.N., Quast L.M. (1997). Spinal Column Deformity and Instability after Lumbar or Thoracolumbar Laminectomy for Intraspinal Tumors in Children and Young Adults. Spine.

[4] McGirt M.J., Chaichana K.L., Atiba A., Bydon A., Witham T.F., Yao K.C., Jallo G.I. (2008). Incidence of spinal deformity after resection of intramedullary spinal cord tumors in children who underwent laminectomy compared with laminoplasty. J. Neurosurg. Pediatr..

[5] De Jonge T., Slullitel H., Dubousset J., Miladi L., Wicart P., Illés T. (2005). Late-onset spinal deformities in children treated by laminectomy and radiation therapy for malignant tumours. Eur. Spine J..

[6] Kothbauer K.F. (2007). Neurosurgical Management of Intramedullary Spinal Cord Tumors in Children. Pediatr. Neurosurg..

[7] Campbell R.M., Smith M.D., Mayes T.C., Mangos J.A., Willey-Courand D.B., Kose N., Pinero R.F., Alder M.E., Duong H.L., Surber J.L. (2003). The Characteristics of Thoracic Insufficiency Syndrome Associated with Fused Ribs and Congenital Scoliosis. J. Bone Jt. Surg.-Am. Vol..

[8] Campbell R.M., Smith M.D., Hell-Vocke A.K. (2004). Expansion thoracoplasty: The surgical technique of opening-wedge thoracostomy. Surgical technique. J. Bone Jt. Surg.-Am. Vol..

[9] Lorenz H.M., Badwan B., Hecker M.M., Tsaknakis K., Groenefeld K., Braunschweig L., Hell A.K. (2017). Magnetically Controlled Devices Parallel to the Spine in Children with Spinal Muscular Atrophy. JBJS Open Access.

[10] Oyoun N.A., Stuecker R. (2014). Bilateral rib-to-pelvis Eiffel Tower VEPTR construct for children with neuromuscular scoliosis: A preliminary report. Spine J..

[11] Lorenz H.M., Braunschweig L., Schiele S., Tsaknakis K., Hecker M.M., Gantner A.S., Hell A.-K. (2018). Surgical Treatment of Spinal Deformities in Young Paraplegic Children with Intraspinal Tumors. Pediatr. Neurosurg..

[12] Samdani A.F., Ranade A., Dolch H.J., Williams R., Hilaire T.S., Cahill P., Betz R.R. (2009). Bilateral use of the vertical expandable prosthetic titanium rib attached to the pelvis: A novel treatment for scoliosis in the growing spine. J. Neurosurg. Spine.

[13] Simon S.L., Auerbach J.D., Garg S., Sutton L.N., Telfeian A.E., Dormans J.P. (2008). Efficacy of Spinal Instrumentation and Fusion in the Prevention of Postlaminectomy Spinal Deformity in Children with Intramedullary Spinal Cord Tumors. J. Pediatr. Orthop..

[14] Giglio C.A., Volpon J.B. (2007). Development and evaluation of thoracic kyphosis and lumbar lordosis during growth. J. Child. Orthop..

[15] Paulino A.C., Fowler B.Z. (2005). Risk factors for scoliosis in children with neuroblastoma. Int. J. Radiat. Oncol..

[16] Fraser R., Paterson D., Simpson D. (1977). Orthopaedic aspects of spinal tumors in children. J. Bone Jt. Surg. Br. Vol..

[17] McGirt M.J., Constantini S., Jallo G.I. (2008). Correlation of a preoperative grading scale with progressive spinal deformity following surgery for intramedullary spinal cord tumors in children. J. Neurosurg. Pediatr..

[18] Arikoski P., Komulainen J., Riikonen P., Parviainen M., Jurvelin J.S., Voutilainen R., Kröger H. (1999). Impaired Development of Bone Mineral Density During Chemotherapy: A Prospective Analysis of 46 Children Newly Diagnosed with Cancer. J. Bone Miner. Res..

[19] Butler M.S., Robertson W.W., Rate W., D’Angio G.J., Drummond D.S. (1990). Skeletal sequelae of radiation therapy for malignant childhood tumors. Clin. Orthop..

[20] Dörr W., Kallfels S., Herrmann T. (2013). Late bone and soft tissue sequelae of childhood radiotherapy. Relevance of treatment age and radiation dose in 146 children treated between 1970 and 1997. Strahlenther. Onkol..

[21] Anakwenze O., Auerbach J.D., Buck D.W., Garg S., Simon S.L., Sutton L.N., Sponseller P.D., Dormans J.P. (2011). The Role of Concurrent Fusion to Prevent Spinal Deformity after Intramedullary Spinal Cord Tumor Excision in Children. J. Pediatr. Orthop..

[22] Halawi M.J., Lark R.K., Fitch R.D. (2015). Neuromuscular Scoliosis: Current Concepts. Orthopedics.

[23] Bartonek A., Saraste H., Eriksson M., Knutson L., Cresswell A.G. (2002). Upper body movement during walking in children with lumbo–sacral myelomeningocele. Gait Posture.

[24] Baniasad M., Farahmand F., Arazpour M., Zohoor H. (2018). Role and Significance of Trunk and Upper Extremity Muscles in Walker-Assisted Paraplegic Gait: A Case Study. Top. Spinal Cord Inj. Rehabil..

[25] Tsirikos A.I., Saifuddin A., Noordeen M.H. (2005). Spinal deformity in neurofibromatosis type-1: Diagnosis and treatment. Eur. Spine J..

[26] Cheung K.M.-C., Cheung J.P.-Y., Samartzis D., Mak K.-C., Wong Y.-W., Cheung W.-Y., Akbarnia B.A., Luk K.D.-K. (2012). Magnetically controlled growing rods for severe spinal curvature in young children: A prospective case series. Lancet.

[27] Gantner A.S., Braunschweig L., Tsaknakis K., Lorenz H.M., Hell A.K. (2018). Spinal deformity changes in children with long-term vertical expandable prosthetic titanium rib treatment. Spine J..

[28] Hell A.K., Hefti F., Campbell R.M. (2004). Treatment of congenital scoliosis with the vertical expandable prosthetic titanium rib implant. Orthopade.

[29] Angevine P.D., McCormick P.C. (2007). Spinal deformity and pediatric intramedullary spinal cord tumors. J. Neurosurg. Pediatr..

[30] Cobb J. (1948). Outline for the study of scoliosis. Instr. Course Lect..

[31] Allison N. (2010). The Classic: The Iliac Apophysis: An Invaluable Sign in the Management of Scoliosis. Clin. Orthop. Relat. Res..

